# The Inhibitory Effect of Alisol A 24-Acetate from *Alisma canaliculatum* on Osteoclastogenesis

**DOI:** 10.1155/2015/132436

**Published:** 2015-07-27

**Authors:** Kwang-Jin Kim, Alain Simplice Leutou, Jeong-Tae Yeon, Sik-Won Choi, Seong Hwan Kim, Sung-Tae Yee, Kyung Hee Choi, Sang-Jip Nam, Young-Jin Son

**Affiliations:** ^1^Department of Pharmacy, Sunchon National University, Suncheon, Jeonnam 540-742, Republic of Korea; ^2^Department of Chemistry and Nano Science, Global Top 5 Program, Ewha Womans University, Seoul 120-750, Republic of Korea; ^3^Research Institute of Basic Science, Sunchon National University, Suncheon 540-742, Republic of Korea; ^4^Laboratory of Translational Therapeutics, Pharmacology Research Center, Division of Drug Discovery Research, Korea Research Institute of Chemical Technology, Daejeon 305-600, Republic of Korea

## Abstract

Osteoporosis is a disease that decreases bone mass. The number of patients with osteoporosis has been increasing, including an increase in patients with bone fractures, which lead to higher medical costs. Osteoporosis treatment is all-important in preventing bone loss. One strategy for osteoporosis treatment is to inhibit osteoclastogenesis. Osteoclasts are bone-resorbing multinucleated cells, and overactive osteoclasts and/or their increased number are observed in bone disorders including osteoporosis and rheumatoid arthritis. Bioactivity-guided fractionations led to the isolation of alisol A 24-acetate from the dried tuber of *Alisma canaliculatum*. Alisol A 24-acetate inhibited RANKL-mediated osteoclast differentiation by downregulating NFATc1, which plays an essential role in osteoclast differentiation. Furthermore, it inhibited the expression of DC-STAMP and cathepsin K, which are related to cell-cell fusion of osteoclasts and bone resorption, respectively. Therefore, alisol A 24-acetate could be developed as a new structural scaffold for inhibitors of osteoclast differentiation in order to develop new drugs against osteoporosis.

## 1. Introduction

Bone is a living, dynamic tissue that is constantly remodeled in the process of bone turnover. Bone health in adults depends on the synchronized performance of bone-resorbing osteoclasts and bone-forming osteoblasts that function together on the bone surface [1]. Bone remodeling is important in vertebrates to maintain bone volume and calcium homeostasis [2]. An imbalance in the activities of bone-resorbing osteoclast cells and bone-depositing osteoblast cells upon aging or reaching menopause leads to osteoporosis [3]. Osteoporosis, Paget's disease, and rheumatoid arthritis are the result of overactive osteoclasts, which resorb bone [4]. This disorder has been increasing in frequency along with the increase in life expectancy [5].

Osteoclasts are tissue-specific macrophage polykaryons created by the differentiation of monocyte/macrophage precursor cells at or near the bone surface [6]. These cells have essential roles in the balance of skeletal homeostasis. Osteoclast differentiation from bone marrow-derived macrophages (BMMs) is needed for receptor activator of nuclear factor-*κ*B ligand (RANKL), which is known to play an important role in osteoclast development [7]. The nuclear factor of activated T cells c1 (NFATc1), noted master transcription factor for osteoclast differentiation, is induced by RANKL [8]. NFATc1 promotes the expression of osteoclast differentiation-related factors including tartrate-resistant acid phosphatase (TRAP), cathepsin K, and dendritic cell-specific transmembrane protein (DC-STAMP) [9–11].

Plants are valuable sources of medicinal compounds with a broad range of biological activity. Approximately 25 to 50% of current pharmaceuticals are derived from plants [12]. Traditional oriental herbal medicines have been reevaluated by clinicians [13] because these medicines have fewer side effects and are more suitable for long-term use compared to chemically synthesized medicines [14].

*Alisma canaliculatum*, a member of the plant family Alismataceae, is a herb commonly used in traditional Korean medicine.* Rhizoma Alismatis*, a dried tuber of* A. canaliculatum*, is the main medicinal part of the plant.* A. canaliculatum* has diuretic hepatoprotective, antitumor, and antibacterial effects [15]. Previous phytochemical and pharmacological investigations of this plant reported the isolation of protostane- and seco-protostane-type triterpenes [16] such as alisols A, B, and C, alisol A 24-acetate, alisol B 23-acetate, alisol C 23-acetate, and alismalactone 23-acetate, and guaiane-type sesquiterpenes [17] such as alismols A and B, sulfoorientalol A, and orientatols AB, C, E, and F.

**Figure 1:**
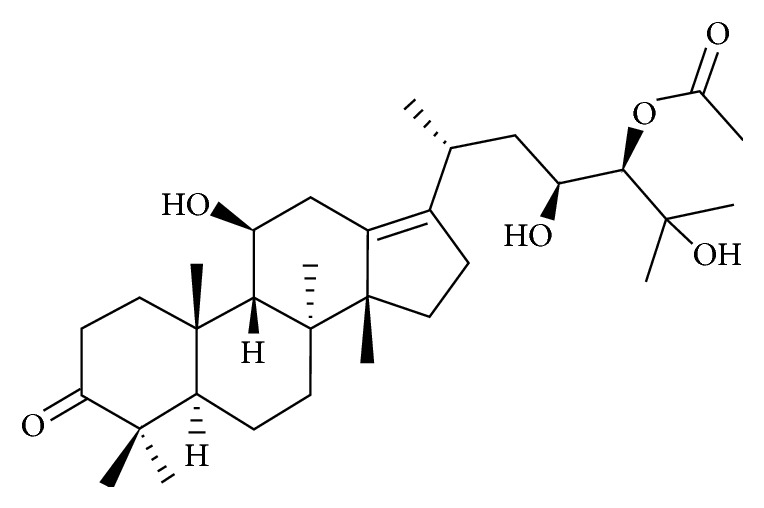
Molecular structure of alisol A 24-acetate.

In our ongoing investigation of biologically active compounds from natural products, the dried rhizomes of* A. canaliculatum* were examined, and bioactivity-guided fractionations and HPLC yielded a triterpenoid, alisol A 24-acetate (Figure 1).

Herein, we report the isolation and the biological activities of alisol A 24-acetate.

## 2. Materials and Methods

### 2.1. Reagents

Recombinant mouse receptor activator of nuclear factor-*κ*B ligand (RANKL) and recombinant mouse macrophage-colony stimulating factor (M-CSF) were purchased from R&D Systems (MN). Cell culture medium, fetal bovine serum (FBS), and penicillin/streptomycin were purchased from Invitrogen Life Technologies (NY). The CCK-8 assay kit was obtained from Dojindo Molecular Technologies (ML). All reagents used in the reverse transcription (RT) and real-time PCR master mix were from Enzynomics (KR). NFATc1 monoclonal and actin polyclonal antibody were from Santa Cruz Biotechnology (CA, USA).

### 2.2. Plant Material

*Alisma canaliculatum* was purchased from Dongbu plant market in Suncheon in the South Sea in Korea.

### 2.3. Extraction and Isolation

The dried rhizomes of* Alisma canaliculatum* (wet weight, 1.2 kg) were minced and extracted with ethanol at room temperature for five days; the ethanol was concentrated under vacuum and then partitioned between EtOAc and H_2_O (1 : 1). The EtOAc-soluble layer was concentrated under vacuum to give 18.0 g, which was subjected to silica gel (0.040–0.063 mm) column chromatography using a stepwise gradient with solvents of increasing polarity, from 100% CH_2_Cl_2_ to 100% MeOH. The fraction containing triterpenoid mixtures eluting with 2% CH_2_Cl_2_ in MeOH was further purified by RP-HPLC [Phenomenex Luna RP-C18(2), 5 *μ*m, 250 × 10 mm, 2.5 mL/min] using an isocratic solvent system with 85% acetonitrile in H_2_O to afford alisol A 24-acetate (**1**, 7.0 mg, *t*_*R*_ 14 min).

### 2.4. Alisol A 24-Acetate (1)

^1^H NMR (CDCl_3_, 700 MHz):*δ*H 4.65 (1H, s, H-24), 3.89 (2H, overlapped, H-11 and H-23), 2.81 (2H, dd,* J* = 13.8, 5.9 Hz H-12), 2.68 (1H, m H-20), 2.35 (2H, ddd,* J* = 15.5, 9.6, 3.3 Hz, H-2), 2.25 (1H, m, Ha-1), 2.20 (3H, s,* -*COCH_3_), 2.15 (1H, m, Hb-1), 2.16 (2H, m, H-16), 2.10 (1H, m, H-5), 2.02 (2H, m, H-7), 1.89 (1H, m, H-15a), 1.74 (1H, d,* J* = 10.8 Hz, H-9), 1.45 (1H, m, H-6a), 1.39 (1H, m, H-6b), 1.38 (2H, m, H-22), 1.36 (1H, m, H-15b), 1.30 (3H, s, H-27), 1.16 (3H, s, H-26), 1.15 (3H, s, H-30), 1.07 (3H, d,* J* = 11.0 Hz, H-21), 1.06 (3H, s, H-28), 1.00 (3H, s, H-18), 0.99 (3H, s, H-19), 0.98 (3H, s, H-29); ^13^C NMR (175 MHz, CDCl_3_): *δ*C 220*.5* (qC, C-3), 171.5 (-COCH_3_), 138.3 (qC, C-13), 135.5 (qC, C-17), 78.6 (CH, C-24), 73.9 (qC, C-25), 70.0 (CH, C-11), 69.0 (CH, C-23), 57.0 (qC, C-14), 49.6 (CH, C-9), 48.5 (CH, C-5), 47.0 (qC, C-4), 40.5 (qC, C-8), 39.7 (CH_2_, C-22), 36.9 (qC, C-10), 34.5 (CH_2_, C-12), 34.3 (CH_2_, C-7), 33.8 (CH_2_, C-2), 30.9 (CH_2_, C-1), 30.5 (CH_2_, C-15), 29.6 (CH_3_, C-28), 29.1 (CH_2_, C-16), 27.9 (CH, C-20), 27.5 (CH_3_, C-26), 26.6 (CH_3_, C-27), 25.7 (CH_3_, C-19), 24.1 (CH_3_, C-30), 23.2 (CH_3_, C-18), 20.1 (-COCH_3_), 20.1 (CH_3_, C-29), 20.1 (CH_3_, C-21), 20.0 (CH_2_, C-6); LCMS *m*/*z*: 515 [M-H_2_O+H]^+^, 497 [M-2H_2_O+H]^+^.

### 2.5. Osteoclast Differentiation

This study was carried out in strict accordance with the recommendations outlined in the Standard Protocol for Animal Study from the Korea Research Institute of Chemical Technology (KRICT; Permit number 2012-7D-02-01). The protocol (ID number 7D-M1) was approved by the Institutional Animal Care and Use Committee of KRICT (IACUC-KRICT). All efforts were made to minimize the suffering of animals. Bone marrow cells (BMCs) were collected from femur and tibia of 5-6-week-old male ICR mice by flushing femurs and tibias with *α*-MEM supplemented with antibiotics. BMCs were cultured with M-CSF (10 ng/mL) in *α*-MEM containing 10% fetal bovine serum (FBS) and antibiotics in a culture dish for 1 day. Nonadherent BMCs were cultured for 3 days in a Petri dish in M-CSF (30 ng/mL), and the adherent cells were used as bone marrow-derived macrophages (BMMs). For the formation of osteoclasts, BMMs were cultured with RANKL (10 ng/mL) and M-CSF (30 ng/mL) in the presence or absence of alisol A 24-acetate for 4 days. The culture medium was changed once per three days. Osteoclast formation was assessed by TRAP (tartrate-resistant acid phosphatase) staining.

### 2.6. TRAP Staining Assay

Cells were fixed with 3.7% formalin for 5 min, permeabilized with 0.1% Triton X-100 for 10 min, and stained with TRAP solution (Sigma-Aldrich, MO, USA) for 10 min. TRAP^+^-MNCs (3 ≤ nuclei) were counted as multinucleated osteoclasts.

### 2.7. Cytotoxicity Assay

The BMMs were cultured with M-CSF (30 ng/mL) at a density of 1 × 10^4^ cells/well on 96-well plates in the presence of alisol A 24-acetate (indicated concentration) for 3 days. The cells were incubated for 3 hours in *α*-MEM containing 10% CCK-8 reagent. The optical density (OD) values were measured at 450 nm.

### 2.8. Real-Time PCR Analysis

Primers were chosen with Primer3 design program [18]. The primer sets used in this study are shown in Table 1. Total RNA was isolated from cells with TRIzol reagent according to the manufacturer's protocol. First-strand cDNA was synthesized with 0.5 *μ*g of total RNA, 1 *μ*M oligo-dT18 primer, and the M-MLV cDNA synthesis kit (Enzynomics, KR) according to the manufacturer's protocol. SYBR green-based QPCR was performed with the Stratagene Mx3000P real-time PCR system and TOPrealTM qPCR 2X PreMIX (Enzynomics, KR), with the first-strand cDNA diluted 1 : 10 and 20 pmol of primers according to the manufacturer's protocol. The polymerase was activated at 95°C for 10 minutes, followed by 40 cycles of 94°C for 30 s (denaturation), 60°C for 30 s (annealing), and 72°C for 30 s (extension). This was followed by the generation of PCR-product temperature-dissociation curves (also called melting curves) at 95°C for 1 min, 55°C for 30 s, and 95°C for 30 s. All reactions were run in triplicate, and data were analyzed by the 2^−ΔΔCT^ method [19]. Glyceraldehyde-3-phosphate dehydrogenase (GAPDH) was used as an internal standard.

### 2.9. Western Blotting Analysis

Cells were incubated in lysis buffer (50 mM Tris-HCl, 150 mM NaCl, 5 mM ethylenediaminetetraacetic acid (EDTA), 1% Triton X-100, 1 mM sodium fluoride, 1 mM sodium vanadate, and 1% deoxycholate, 1 : 1000 proteinase inhibitor) for 30 minutes on ice. Cell lysates were separated by SDS-PAGE and transferred to a polyvinylidene difluoride membrane (Millipore). The membranes were washed with TBST (10 mM Tris-HCl pH 7.5, 150 mM NaCl, and 0.1% Tween 20) and incubated in blocking buffer (5% nonfat milk in TBST) for 1 hour at room temperature. The membranes were incubated with anti-NFATc1 (1 : 500) and anti-actin (1 : 1000) overnight. After three 30 min wash, the membranes were incubated with secondary antibody conjugated to horseradish peroxidase for 2 hours at room temperature and then washed three times for 30 min. Specific bands were visualized by chemiluminescence using the LAS-3000 luminescent image analyzer (Fuji Photo Film Co., Ltd., Japan).

### 2.10. All Quantitative Values Are Presented as Mean ± SD

Each experiment was performed three to five times, and the results from one representative experiment are shown. Statistical differences were analyzed using Student's *t*-test of Microsoft Excel. The *P* values were described by the comparison between the control and one of the test groups (^*∗*^ *P* < 0.05; ^*∗∗*^ *P* < 0.01; ^*∗∗∗*^ *P* < 0.001). A value of *P* < 0.05 was considered significant.

## 3. Results

### 3.1. Alisol A 24-Acetate Inhibited the Differentiation of BMMs by RANKL

**Figure 2:**
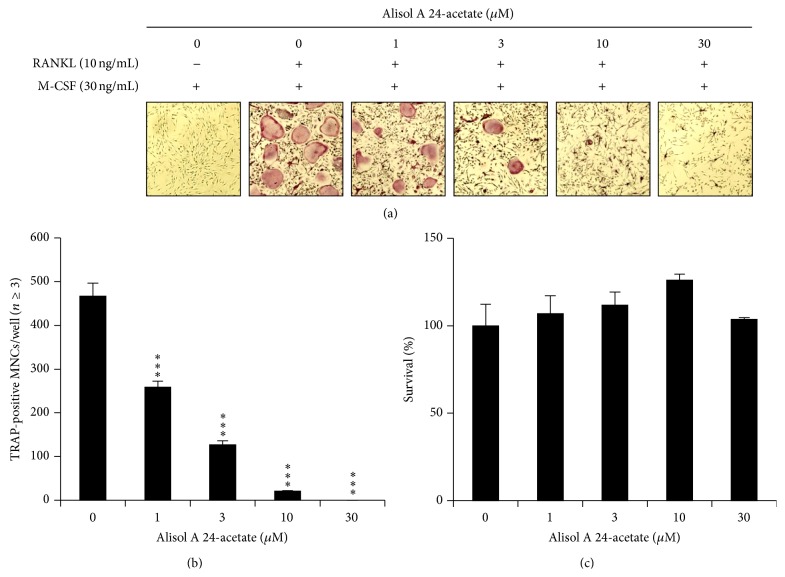
Effects of alisol A 24-acetate on osteoclastogenesis. (a) BMMs prepared from bone marrow cells were cultured for 4 days with RANKL (10 ng/mL) and M-CSF (30 ng/mL) in the presence of the indicated concentrations of alisol A 24-acetate. Cells were fixed in 3.7% formalin, permeabilized in 0.1% Triton X-100, and stained for TRAP, a marker enzyme of osteoclasts. (b) TRAP-positive multinuclear cells with three or more nuclei were counted as osteoclasts. ^*∗∗*^ *P* < 0.01; ^*∗∗∗*^ *P* < 0.001 (*n* = 3). (c) Effect of alisol A 24-acetate on the viability on BMMs was evaluated by CCK-8 assay.

To determine the effect of alisol A 24-acetate on osteoclast differentiation, alisol A 24-acetate was added during osteoclast differentiation with RANKL (10 ng/mL) and M-CSF (30 ng/mL). The addition of alisol A 24-acetate inhibited the differentiation of BMMs into osteoclasts (Figure 2(a)). In addition, the number of TRAP-positive multinucleated cells (3 ≤ nuclei) was significantly decreased in a dose-dependent manner by alisol A 24-acetate (Figure 2(b)). Osteoclasts were completely inhibited at a concentration of 10 *μ*M alisol A 24-acetate. These results implied that alisol A 24-acetate could inhibit RANKL-induced osteoclastogenesis.

### 3.2. The Cytotoxic Effect of Alisol A 24-Acetate

The cytotoxicity of alisol A 24-acetate during osteoclast differentiation was measured by CCK-8 assay. BMMs were incubated in the presence of M-CSF (30 ng/mL) and DMSO (vehicle) or alisol A 24-acetate for 3 days. Alisol A 24-acetate had no cytotoxic effects at the indicated concentration (Figure 2(c)). These results suggested that osteoclastogenesis suppression by alisol A 24-acetate was not due to toxic effects on BMMs.

### 3.3. Alisol A 24-Acetate Inhibited RANKL-Induced mRNA Expression of Osteoclast-Specific Genes

**Figure 3:**
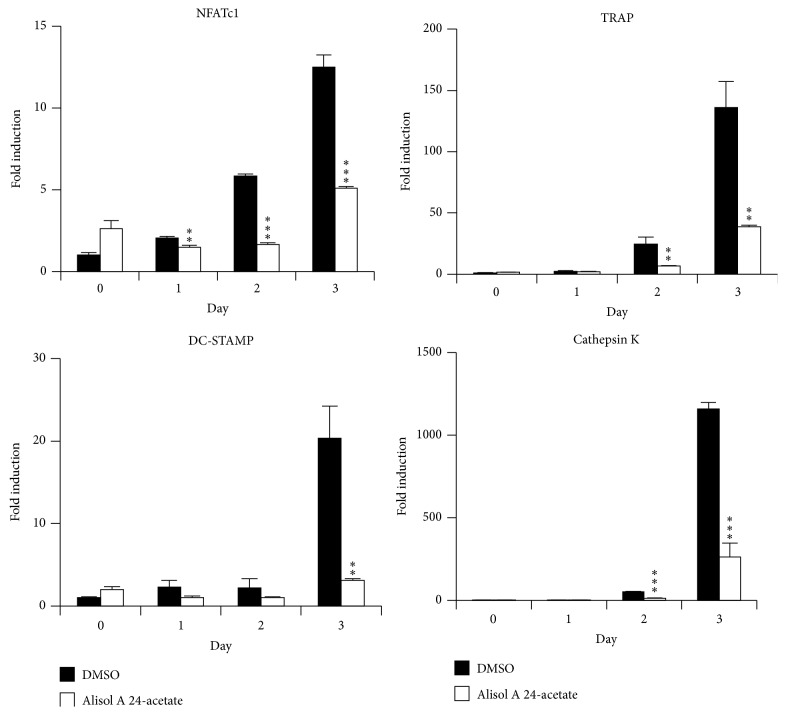
Alisol A 24-acetate decreased NFATc1 transcriptional expression by RANKL stimulation. BMMs were pretreated with vehicle (DMSO) or alisol A 24-acetate (10 *μ*M) for 30 minutes and then stimulated with RANKL (10 ng/mL) for the indicated number of days. Expressed mRNA levels were analyzed by real-time PCR compared with the vehicle control. ^*∗∗*^ *P* < 0.01; ^*∗∗∗*^ *P* < 0.001 (*n* = 3).

We investigated mRNA expression of osteoclast-specific genes in osteoclast differentiation by real-time PCR. Expressed mRNA levels of NFATc1, TRAP, DC-STAMP, and cathepsin K were analyzed compared with the control (DMSO) for 3 days. Alisol A 24-acetate significantly suppressed mRNA expression of transcription factors such as NFATc1. Furthermore, it decreased osteoclast-related molecules including TRAP, DC-STAMP, and cathepsin K (Figure 3).

### 3.4. Alisol A 24-Acetate Inhibited RANKL-Induced Protein Expression of NFATc1

**Figure 4:**
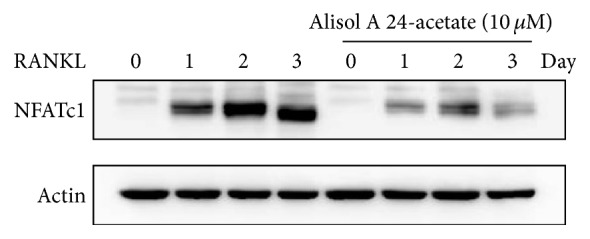
Alisol A 24-acetate inhibits RANKL-induced NFATc1 expression. BMMs were pretreated with alisol A 24-acetate (10 *μ*M) for 1 h and then stimulated with RANKL (10 ng/mL) for the indicated time. Cell lysates were resolved by SDS-PAGE, and western blotting was performed with anti-NFATc1 and anti-actin antibodies as indicated.

The inhibitory effect of alisol A 24-acetate on the translational expression of NFATc1, a master regulator of osteoclast differentiation, was evaluated by western blot analysis. Protein expression of NFATc1 was significantly increased by RANKL without alisol A 24-acetate but was dramatically inhibited by alisol A 24-acetate (Figure 4). This result indicated that alisol A 24-acetate could inhibit the translational expression of NFATc1 and suppress osteoclastogenesis.

## 4. Discussion

Osteoporosis is a bone disease characterized by low bone mass and structural deterioration of bone tissue. Osteoporosis causes nearly nine million new osteoporotic fractures annually worldwide [20]. Low bone mineral density (BMD) is a major cause of bone fracture. BMD is affected by bone-resorbing osteoclasts and bone-forming osteoblasts.

Bone remodeling is an important process in sustaining healthy bones. It is carried out by osteoblasts and osteoclasts. The balance between osteoblastic bone formation and osteoclastic bone resorption is crucial for bone homeostasis. Generally, bone disorders such as osteoporosis and rheumatoid arthritis involve overactive osteoclasts and/or their increased number. Osteoclasts, derived from pluripotent hematopoietic stem cells, are bone-resorbing multinucleated cells [6, 21, 22]. RANKL, an osteoclasts differentiation factor, is related to the TNF superfamily and expressed by stromal cells in bone marrow and osteoblasts [23, 24]. It contains a C-terminal receptor-binding domain and a transmembrane domain and binds to its receptor, RANK, which is expressed on osteoclasts [25]. RANKL/RANK binding activates NFATc1, which regulates many osteoclast-specific genes, such as cathepsin K, TRAP, and DC-STAMP [24, 25]. TRAP is the principal cytochemical marker for osteoclasts [26], DC-STAMP plays an essential role in cell-cell fusion of osteoclasts [11, 27], and cathepsin K is a major protease in bone resorption [28].

Here, we tested the effect of alisol A 24-acetate on osteoporosis, specifically RANKL-mediated osteoclast differentiation. The alisol A 24-acetate, isolated from the dried tuber of* Alisma canaliculatum*, completely inhibited osteoclast differentiation and had no cytotoxic effects at concentrations over 10 *μ*M. These results suggested that the alisol A 24-acetate has antiosteoclastogenic activity without cytotoxicity to BMMs. As mentioned earlier, the expression of NFATc1 is the key factor in osteoclastogenesis. So we investigated the transcriptional expression level of NFATc1 and some osteoclast-specific genes for osteoclastogenesis. The mRNA expression of NFATc1 was inhibited by alisol A 24-acetate. Furthermore, the mRNA expression levels of osteoclast-specific genes for osteoclast differentiation such as TRAP, DC-STAMP, and cathepsin K were significantly reduced by alisol A 24-acetate. Thus, alisol A 24-acetate inhibited the signal cascade from RANKL/RANK binding to NFATc1, and osteoclast differentiation was inhibited because of the inhibitive mechanism of alisol A 24-acetate. Moreover, alisol A 24-acetate blocked cell-cell fusion of osteoclasts by inhibiting the expression of DC-STAMP. The decreased expression of DC-STAMP and cathepsin K was related to the decreased expression of NFATc1 [29, 30]. We confirmed the inhibition of alisol A 24-acetate on the translational expression of NFATc1 by western blotting. Like the transcriptional inhibition of NFATc1, the translational expression of NFATc1 was strongly inhibited by alisol A 24-acetate. Our results suggest that alisol A 24-acetate may be a potential therapeutic molecule for bone disorders and could be utilized as a new structural scaffold for inhibitors of osteoclast differentiation.

## 5. Conclusions

This is the first report of alisol A 24-acetate, isolated from* Alisma canaliculatum*, and its antiosteoclastogenic activity. Alisol A 24-acetate inhibited RANKL-induced osteoclast differentiation by downregulating NFATc1, a master factor for osteoclast differentiation, without cytotoxicity and also inhibited the expression of DC-STAMP and cathepsin K. Therefore, alisol A 24-acetate could be used as a scaffold for the development of a new osteoporosis drug.

## Figures and Tables

**Table 1:** Primer sequences used in this study.

Target gene	Forward (5′-3′)	Reverse (5′-3′)
NFATc1	GGGTCAGTGTGACCGAAGAT	GGAAGTCAGAAGTGGGTGGA
TRAP	GATGACTTTGCCAGTCAGCA	ACATAGCCCACACCGTTCTC
Cathepsin K	GGCCAACTCAAGAAGAAAAC	GTGCTTGCTTCCCTTCTGG
DC-STAMP	CCAAGGAGTCGTCCATGATT	GGCTGCTTTGATCGTTTCTC
GAPDH	ACCACAGTCCATGCCATCAC	TCCACCACCCTGTTGCTGTA
